# Case study to assess the safety of irreversible electroporation near the heart

**DOI:** 10.1186/s40064-015-0828-7

**Published:** 2015-02-11

**Authors:** Katsutoshi Sugimoto, Fuminori Moriyasu, Hirohito Takeuchi, Mayumi Ando, Takatomo Sano, Toshifumi Mori, Yoshihiro Furuichi, Yoshiyuki Kobayashi, Ikuo Nakamura

**Affiliations:** Department of Gastroenterology and Hepatology, Tokyo Medical University, 6-7-1 Nishishinjuku, Shinjuku-ku, Tokyo 160-0023 Japan

**Keywords:** Irreversible electroporation, Arrhythmia, Complication, Tumor, Metastasis, Liver

## Abstract

**Introduction:**

Irreversible electroporation (IRE) is a promising technique for the focal treatment of soft tissue tumors. Even though the local application of an excessive electric field is a potential cause of cardiac arrhythmias, initial clinical studies have shown that IRE is generally safe when cardiac gating is employed.

**Case description:**

In this case report, we observed an episode of ventricular extrasystoles without hemodynamic changes during which time the synchronization device failed to operate properly, with pulses delivered not in the absolute refractory period but in the relative refractory period.

**Discussion and evaluation:**

At present, persons performing IRE must keep in mind that there is a small but real risk of synchronization failure even when a cardiac synchronization device is used.

**Conclusion:**

It is advisable to err on the side of caution when treating lesions near the heart.

## Introduction

Irreversible electroporation (IRE) is a new non-thermal local ablative treatment procedure in which irreversible permeabilization of the cell membrane lipid bilayer is induced by the creation of nanopores, resulting in cell death (Davalos et al. 2005). IRE may offer two major potential advantages over commonly employed radiofrequency (RF) thermal ablation approaches for the treatment of hepatic lesions.

The first advantage is that, unlike RF ablation, IRE relies on electrical energy rather than thermal energy to induce cell death, and it appears to be unaffected by nearby vessels (Rubinsky et al. 2007). This suggests that IRE may be more effective for the treatment of areas where tumor cells are in close proximity to large vessels (i.e., no “heat-sink effect”). The second advantage is that IRE may improve safety in the vicinity of heat-sensitive structures such as blood vessels, nerves, bile ducts, and the gastrointestinal tract (Cannon et al. 2013; Schoellnast et al. 2013a; Choi et al. 2014; Schoellnast et al. 2013b). Given its distinctive characteristics, IRE has the potential to become an alternative method for the ablation of solid tumors.

However, specific precautions are required because the delivery of powerful electrical pulses to human tissue has the potential to cause fatal cardiac arrhythmias (Ball et al. 2010). Cardiac screening and synchronized pulsing are therefore absolutely imperative (Deodhar et al. 2011). In an initial clinical study in which cardiac synchronization was employed, only atrial arrhythmias occurred in 4 cases, and these resolved spontaneously or within 24 hours after therapy (Scheffer et al. 2014). Accordingly, initial clinical studies have concluded that IRE may be safe when such measures are taken (Scheffer et al. 2014).

Nonetheless, specific guidelines for the use of IRE (i.e., how close to the heart IRE can safely be performed even with the use of cardiac synchronization) have not yet been established. In this case report, we describe the IRE treatment of metastatic liver lesions located in close proximity to the inferior border of the heart.

## Case report

A 48-year-old man who had recently undergone resection of a moderately differentiated invasive adenocarcinoma of the rectum presented with focal liver lesions measuring 15 mm, 16 mm, and 9 mm in segments 8, 4, and 2 of the liver, respectively, which were newly diagnosed by MR imaging. No other metastatic lesions were detected by PET. He had not a prior medical history of other conditions, including heart disease. He had received one cycle of modified FOLFOX6 chemotherapy (*l*-leucovorin, 5-fluorouracil, and oxaliplatin), but this treatment was halted due to adverse events such as pancytopenia and malaise. He was therefore referred to our department. After discussing treatment options, including resection and ablation, the decision was made to proceed with IRE for these metastases as a part of a clinical trial (UMIN000014522).

We performed ultrasound (US)-guided IRE (NanoKnife; AngioDynamics, Latham, NY, USA) with the patient under general anesthesia with neuromuscular blockade to avoid muscle contraction. All interventions were performed percutaneously using a dedicated US system (Aplio™ 500; Toshiba, Tochigi, Japan) equipped with a 3.75-MHz convex transducer (PVT-385BT; Toshiba, Tochigi, Japan). In addition, to prevent pulse-induced arrhythmias, a preprogrammed commercial ECG trigger monitor (AccuSync 72; AccuSync Medical Research Corporation, Milford, CT, USA) was connected to a five-lead ECG to synchronize pulse delivery within the refractory period of the heart. An external biphasic defibrillator was also prepared and was immediately available for the treatment of ventricular arrhythmias.

Considering the size and shape of each lesion and including a 1-cm tumor-free margin, we determined the number and configuration of electrodes. The voltage was also determined using a standard algorithm (AngioDynamics) based on factors such as the intended size of the ablation zone, the number of probes, the distance between the probes, and the length of the active electrode tip (Thomson et al. 2011). Accordingly, three insulated 15-cm needle electrodes with an exposure length of 20 mm were placed in the outer border of the tumor under US guidance. All needles were placed as parallel to one another as possible to help ensure homogeneous energy delivery.

**Figure 1:**
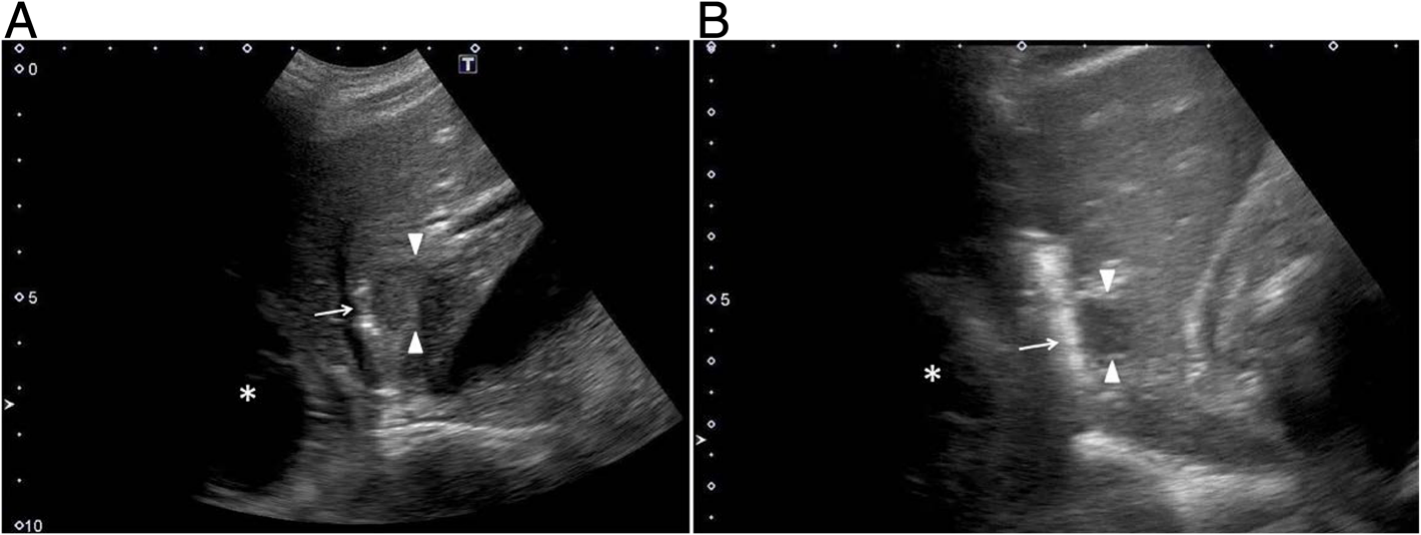
**Illustration of the IRE electrode placed near the heart. A**: Sagittal B-mode US image demonstrates that the IRE electrode (arrow) is placed in the periphery of the hypoechoic lesion (arrowheads) in segment 4. The distance from the electrode to the inferior border of the heart (asterisk) is 1.7 cm based on US measurement. **B**: Sagittal B-mode US image demonstrates that the IRE electrode (arrow) is placed in the periphery of the hypoechoic lesion (arrowheads) in segment 2. The distance from the electrode to the inferior border of the heart (asterisk) is 1.0 cm based on US measurement.

When we treated both lesions located in segments 4 and 2, three IRE electrodes with a separation of 1.5 to 2 cm between each pair of electrodes were used and deployed in a triangular array. One of the three electrodes was placed very close to the heart, with minimum distances from the heart of 17 mm and 10 mm, respectively (Figure 1A, B). After a test pulse at 270 V, 10 tentative pulses at 2250-3000 V (i.e., 1500 V/cm) with a duration of 70 μs were delivered via each electrode pair. After these pulses confirmed adequate conductivity, 80 additional pulses were administered to reach a total of 90 pulses per electrode pair.

**Figure 2:**
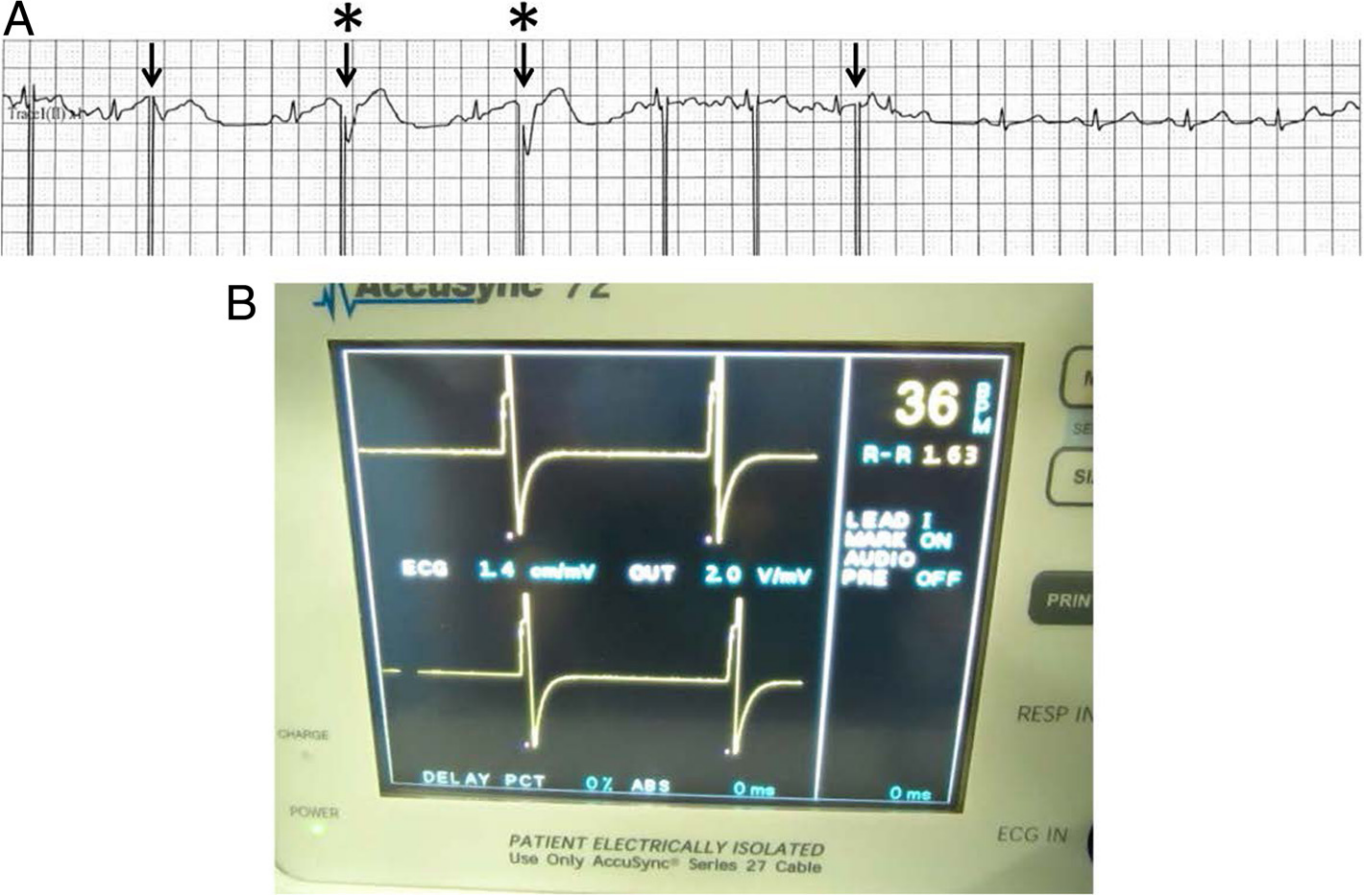
**Electrocardiogram during IRE treatment. A**: Tracing obtained during IRE treatment of the hepatic mass in segment 4 with the synchronization device. The distance from the electrode to the inferior border of the heart is 1.7 cm based on US measurement. IRE pulses are incorrectly delivered on the T wave (arrows), resulting in an episode of ventricular extrasystoles (asterisks). **B**: The synchronization device failed to operate properly during treatment.

**Figure 3:**
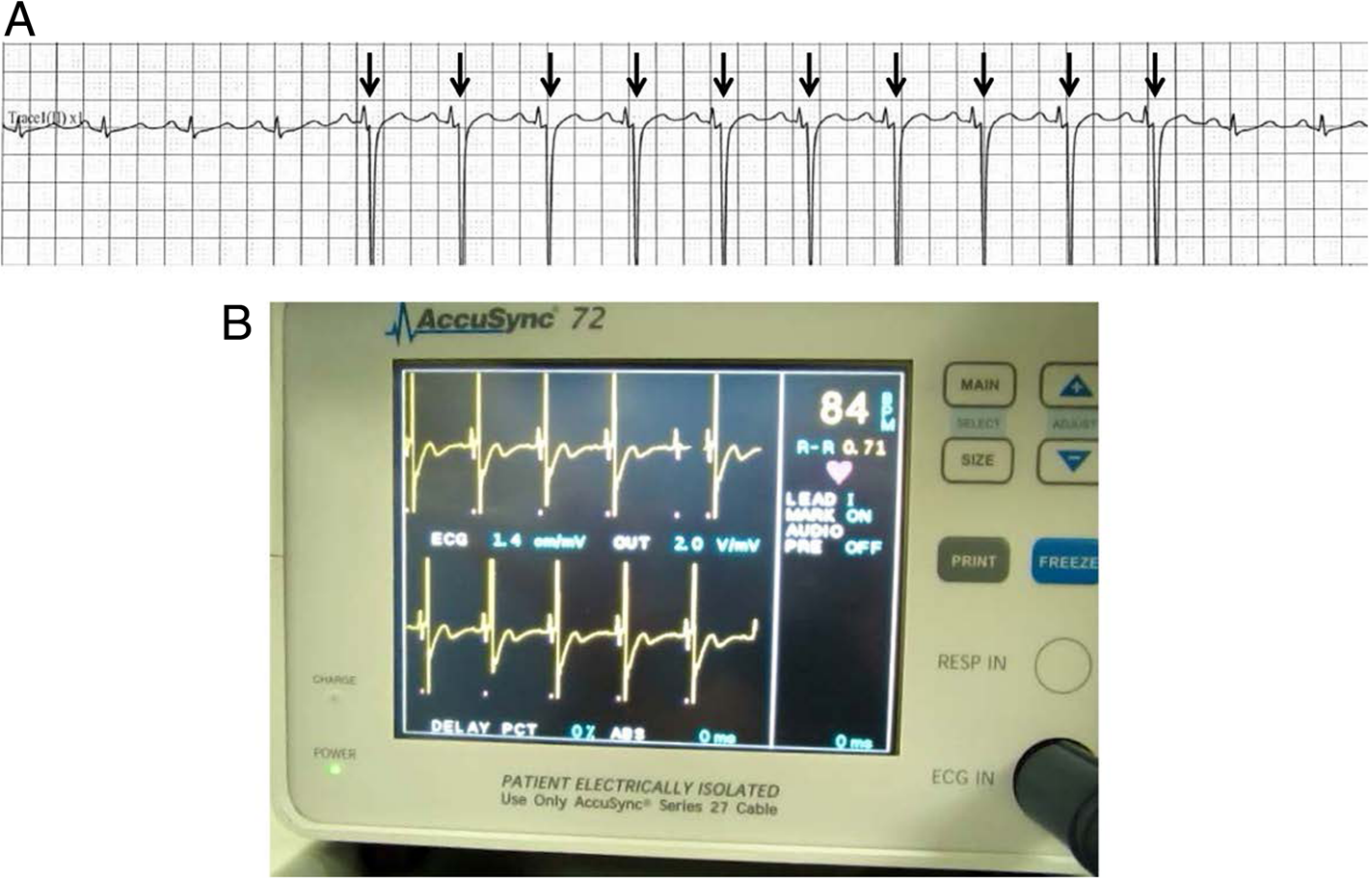
**Electrocardiogram during IRE treatment. A**: Tracing obtained during IRE treatment of the hepatic mass in segment 2 with the synchronization device. The distance from the electrode to the inferior border of the heart is 1.0 cm based on US measurement. IRE pulses are correctly delivered on the R wave (arrows). **B**: The synchronization device operated properly during treatment.

During ablation of the lesion in segment 4, we observed an episode of ventricular extrasystoles without hemodynamic changes during which time the synchronization device failed to operate properly (i.e., unsuccessful synchronization with the R wave), with pulses delivered not in the absolute refractory period but in the relative refractory period (Figure. 2). The arrhythmia ceased immediately without the need to abort the procedure, and IRE was continued without complications. On the other hand, during ablation of the lesion in segment 2, which was closest to the heart, we did not observe any arrhythmias, probably because the synchronization device operated properly (Figure. 3).

## Discussion

In a previous animal study, which was conducted using a 2-electrode configuration, all cells within a radius of 1.7 cm from each applicator were found to show a transient or permanent increase in cell membrane permeability, suggesting that the delivery of IRE pulses within 1.7 cm of the myocardium could induce arrhythmias (Deodhar et al. 2011). Accordingly, the authors of that study stated that when performing IRE ablation, in order to avoid inducing ventricular arrhythmias, the distance from the heart should be greater than 1.7 cm or IRE should be performed in synchronized mode (Deodhar et al. 2011).

The findings of initial clinical studies have also shown that IRE is generally safe when cardiac gating is employed: only minor arrhythmias occurred, with a total incidence of 2% (4 of 194) (Scheffer et al. 2014). However, in our case, we are unable to assert the safety of this method because a number of transient ventricular extrasystoles occurred during IRE. In addition, we found that these ventricular extrasystoles occurred when the synchronization device failed to operate properly (i.e., unsuccessful synchronization with the R wave). As a result, some pulses were delivered during the vulnerable period of the ventricular myocardium, which corresponds to almost the entire T wave as seen on the ECG.

Thomson et al. 2011 reported that their team used a preprogrammed commercial ECG trigger monitor (AccuSync 42; AccuSync Medical Research Corporation) and found it to be unsatisfactory because it lost synchronization during IRE, causing the IRE sequence to be aborted. A more advanced synchronization unit (AccuSync 72; AccuSync Medical Research Corporation) was subsequently used successfully.

Nevertheless, we have occasionally encountered such synchronization failure (Figure. 2B) even though the AccuSync 72 was used. Given this situation, one possible solution is to change the lead pair in each case if synchronization failure is observed. However, this phenomenon is considered to be of critical importance because it could induce ventricular arrhythmias if the distance from the heart to the IRE electrode is less than 1.7 cm. We feel that the manufacturer should make further improvements to the device.

Moreover, it is important to emphasize that a 2-electrode configuration was used in the cited study (Deodhar et al. 2011), whereas a 3-electrode configuration was used in the present study. The distance (i.e., distance of the electrodes from the heart) could be affected by the electrode configuration, the geometry, and the electrical properties of the tissues. It is therefore uncertain whether the distance obtained in this study (i.e., 1.7 cm) is a true threshold value or not. Although a fatal adverse event was fortunately avoided in this case, we feel that it would be wise to avoid IRE treatment if the heart lies in the reversibly permeabilized area until improvements are made to the synchronization device to avoid the possibility of synchronization failure.

In conclusion, persons performing IRE must recognize that there is a possibility of synchronization failure. Even when cardiac synchronization is employed, it is better to err on the side of caution when treating lesions located near the heart.

## Consent

Written informed consent was obtained from the patient for publication of this report and any accompanying images.
